# Influence of Screw Angulation on the Mechanical Properties on a Polyaxial Locking Plate Fixation

**DOI:** 10.3390/bioengineering11101024

**Published:** 2024-10-14

**Authors:** Gabriel Martínez-Fortún, Alejandro Yánez, Alberto Cuadrado

**Affiliations:** Department of Mechanical Engineering, University of Las Palmas de Gran Canaria, 35017 Las Palmas de Gran Canaria, Spain; gabriel.lopez104@alu.ulpgc.es (G.M.-F.); alejandro.yanez@ulpgc.es (A.Y.)

**Keywords:** veterinary medicine, polyaxial screws, angulation, locking plates, cycling test

## Abstract

Polyaxial locking systems are widely used for strategic surgical placement, particularly in cases of osteoporotic bones, comminuted fractures, or when avoiding pre-existing prosthetics. However, studies suggest that polyaxiality negatively impacts system stiffness. We hypothesize that a new plate design, combining a narrow plate with asymmetric holes and polyaxial capabilities, could outperform narrow plates with symmetric holes. Three configurations were tested: Group 1 with six orthogonal screws, and Groups 2 and 3 with polyaxiality in the longitudinal and transverse axes, respectively. A biomechanical model assessed the bone/plate/screw interface under cyclic compression (5000 cycles) and torsion loads until failure. Screws were inserted up to 10° angle. None of the groups showed a significant loss of stiffness during compression (*p* > 0.05). Group 1 exhibited the highest initial stiffness, followed by Group 3 (<29%) and Group 2 (<35%). In torsional testing, Group 1 achieved the most load cycles (29.096 ± 1.342), while Groups 2 and 3 showed significantly fewer cycles to failure (6.657 ± 3.551 and 4.085 ± 1.934). These results confirm that polyaxiality, while beneficial for surgical placement, reduces biomechanical performance under torsion. Despite this, no group experienced complete decoupling of the screw–plate interface, indicating the robustness of the locking mechanism even under high stress.

## 1. Introduction

Long bone fractures such as those of the femur, have been reported as the most common in dogs and cats [1]. Locking plates have gained prominence in the field of veterinary medicine as orthopaedic devices for fracture healing, drawing inspiration from successful precedents established in human orthopaedics [2,3,4].

The inception of internal fixation structures can be traced back to the mid-1800s, gaining significant prominence in the 1960s [5]. The fixation plate serves to establish a robust framework by intricately connecting the two fractured bone ends through screws, thereby fortifying resistance against axial compression, torsional forces, and bending, while simultaneously providing an optimal environment for stable healing. However, the inherent friction between the plate and the bone often leads to deleterious effects on the periosteum and compromises vascular supply, thereby hindering the desired union [6]. In response to this challenge, limited-contact dynamic compression plates were introduced to reduce the contact surface between the plate and bone. Building upon this innovation, locking compression plate (LCP) systems with combination holes were developed as a less invasive, rigid stabilization system [7].

Diverging from dynamic compression plates (DCPs), locking plates exhibit independence from the screw torque to achieve stability at the bone–plate interface. This category of orthopaedic implants attains the load transfer between fracture fragments through angular stability, facilitated by the rigid fixation of threaded screw heads within plate threaded holes, collectively denoted as locked internal fixators (LIFs) [8,9].

The application of polyaxial screws in securing the LCP system to the bone allows for strategic placement, avoiding penetration into the fractured bone area or pre-existing prosthetic structures. Polyaxial locking plate (PLP) systems are also very useful in cases of osteoporotic bones, periarticular and periprosthetic fractures, polytraumatized patients, and high-grade comminuted fractures of long bone diaphyses [10,11].

The commercial availability of PLP systems is highly varied. There are different types of systems that differ mainly by the type of locking system and the width of the plate. Among the existing locking systems, the most common are the point contact locking (Peri-Loc, Smith and Nephew, Memphis, TN, USA), self-tapping locking (VariAx, Stryker, Mahwah, NJ, USA), expansion locking (POLYAX, Biomet, Warsaw, IN, USA), and plug locking (NCB, Zimmer, Warsaw, IN, USA). All these PLP systems have been analyzed in previous studies, thus their weaknesses are known [12,13,14,15].

Previous studies delved into several variable angle locking systems, and all converge on the conclusion that polyaxial locking plates provide surgeons with more options in challenging clinical situations and a reliable avenue for fracture healing. However, caution is necessary when using polyaxiality. Hebert-Davies et al. [16] analyzed the mechanical properties of three different types of locking mechanisms in three configurations (0°, 10°, and 15°) and concluded that polyaxial screws appear to maintain this property up to 10 degrees of angulation. J. E. Tidwell et al. [17] also tested the mechanical properties of a VA locking mechanism with screws placed at various angles (0° to 15°). Their results showed that this polyaxiality has a biomechanical cost subject to loss of stiffness because they found that variable angle systems offer the greatest resistance to rotation when the screw is inserted perpendicular to the plate axis and, furthermore, as the angle of screw polyaxiality increases from 0° to 15°, the resistance to rotation at the screw/plate interface decreases almost linearly. J. Glowacki et al. [18,19] analyzed the mechanical properties from the ChM 5.0 ChLP polyaxial locking system in three configurations (0°, 10°, and 15°) and concluded that polyaxial screws showed a significant reduction in load to failure. J. Kaczmarek et al. [20] examined the influence of screw insertion angle along with the insertion torque on the mechanical properties of a 3.5 mm fixed-angle locking compression plate (LCP) and a 3.5 mm variable-angle polyaxial locking system (PLS), concluding that the 3.5 mm PLS system exhibited a significantly higher performance when the screws were placed at 0 degrees compared to 5, 10, 15, and 20 degrees, regardless of the insertion torque applied.

To our knowledge, only a few studies and guides have been conducted regarding the biomechanical properties of polyaxial locking plates under cyclic torsion loads in veterinary medicine. Ex vivo analyses of the compression and bending properties of polyaxial locking plates under static loads have been performed [21,22,23]. In vivo surgeries have also been researched [24]. Nevertheless, cyclic analysis assumes a paramount importance in elucidating the mechanical properties and wear and tear of orthopaedic devices over time.

Most LCP configurations can be divided into wide plates (with asymmetric holes) and narrow plates (with symmetrical holes) [25]. The hypothesis of this work is that a new concept design, combining narrow plate with asymmetric holes and polyaxial capability, could offer better results than narrow plates with symmetric holes. The analysis has been carried out in various configurations by varying the screw insertion angle through single-cycle and sequentially cyclic fatigue axial compression tests and torsion tests.

## 2. Materials and Methods

### 2.1. Implants

**Figure 1:**
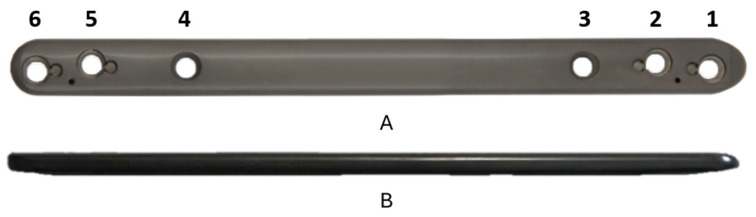
3.5 mm 6-hole polyaxial locking plate (PLP): (**A**) top view; (**B**) lateral view.

A 3.5 mm 6-hole polyaxial locking plate (3.5VA-PLP, Canary Island Institute of Technology S.A., Las Palmas de Gran Canaria, Spain) with an innovative locking system was used. This plate is made from pure titanium (Ti) and has a length of 150 mm. Figure 1 shows the plate model used. Plate holes were listed from top to bottom. The holes closest to the centre of the plate (holes 3 and 4) are aligned with the longitudinal axis of the plate and do not have the possibility of polyaxiality, while the rest of the holes (holes 1, 2, 5, and 6) have a staggered screw–hole arrangement and a polyaxiality of 15° from the midline for screw convergence at the far cortex. Diaphyseal fixation was performed with self-tapping 3.5 mm locking screws, using 40 mm long screws for bicortical fixation. Both the PLP and the screws are products designed by the manufacturer for specific use in the treatment of large bone defects in veterinary medicine.

### 2.2. Speciments

The use of surrogate bone is widely recognized in the mechanical testing field. Plate fixation was evaluated in surrogate specimens of the femoral diaphysis to minimize inter-specimen variability. All tests were performed for non-osteoporotic bone. Cylindrical surrogate bones with 5 mm wall thickness, 30 mm outer diameter, and 265 mm length were used to simulate the material and geometry of natural bone properties (Generic bone model PR0105, Synbone AG, Malans, Switzerland) [26,27,28]. Surrogate bones were cut into equal segments of 132.5 mm length and aligned and fixed with cylindrical bone clamps. All constructs were applied in a standard bridge plating configuration with a 5 mm fracture gap [29,30]. Both the proximal and distal halves of the plate were applied to the surrogate diaphysis with six screws placed in the 1st, 2nd, 3rd, 4th, 5th, and 6th holes from the fracture gap to reflect standard clinical practice. The plate was rigidly fixed to a reinforced surrogate bone with six bicortical screws.

### 2.3. Test Preparation

**Figure 2:**
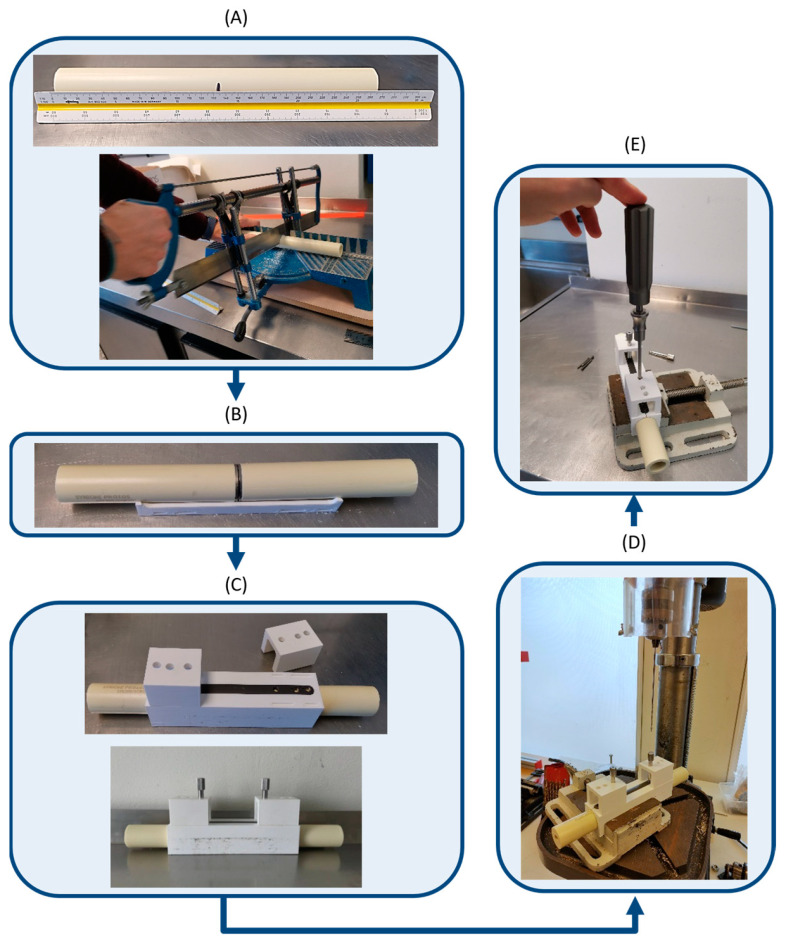
Test specimen assembly process: (**A**) measuring and cutting the surrogate bone; (**B**) gap creation; (**C**) assembly of the fixation and drilling guide; (**D**) drilling the holes for screw insertion; (**E**) insertion and tightening of screws.

The assembly process for each test specimen was performed in accordance with a standardized procedure. Cutting and fixation tools were utilized to ensure process repeatability and to minimize human error during the assembly. The steps for the assembly of each test specimen are as follows:

1. Each surrogate bone is measured, marked at its longitudinal centre, and cut into two equal-length fragments (Figure 2A).
2. The surrogate bone is placed on one of the components of the fixation and drilling guide system, and two steel discs are used to create a 5 mm gap simulating a fracture (Figure 2B).
3. The remaining components of the fixation and drilling guide are assembled to position the polyaxial plate and surgical drill guides (Figure 2C).
4. A vertical column drill, which allowed for adjustment of the drilling angle at the base, is used to drill the holes for screw insertion (Figure 2D).
5. The screws are inserted and tightened into the previously drilled holes using a torque wrench provided by the manufacturer of the polyaxial plates maintaining the plate–bone assembly within the fixation and drilling guide (Figure 2E).

### 2.4. Testing Configurations

Cyclic tests have been divided into three configurations for PLP. The differences among configurations are based on the screw angle and screw insertion plane. In all groups, the plates were filled with three bicortical locking screws on each side of the fracture.

**Figure 3:**
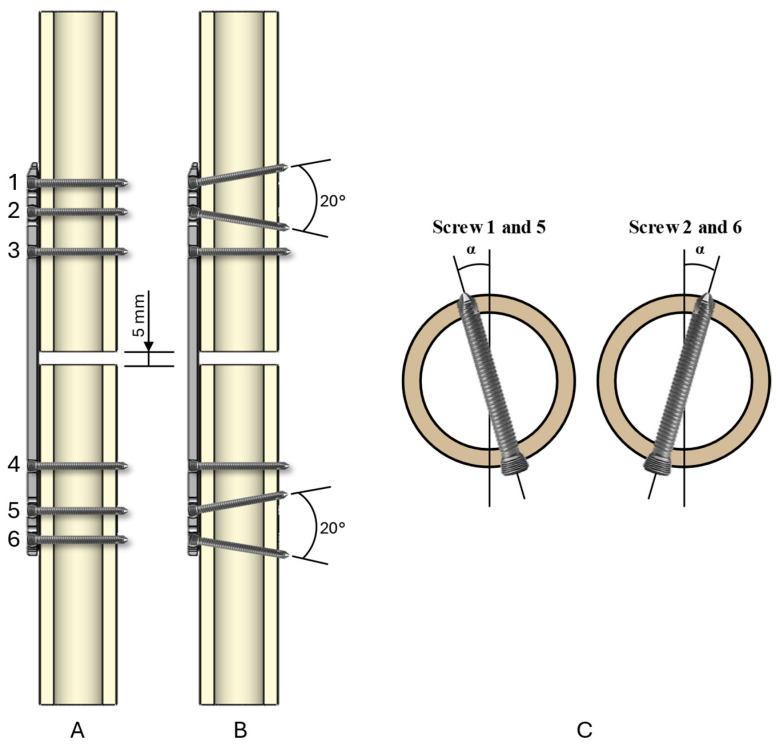
Layout of the three-screw configurations for (**A**) first configuration, drilling angle equal to 0° (Group 1); (**B**) second configuration, drilling angle equal to 10° (Group 2); (**C**) third configuration, 10° off transverse plate plane (Group 3).

For the first configuration, Group 1, the screws were inserted orthogonally to the longitudinal and transverse plane of the plate (Figure 3A). For the second configuration, Group 2, the screws 1, 2, 5, and 6 were inserted polyaxially at a 10° angle off the longitudinal plane (Figure 3B). For the third configuration, Group 3, the screws 1, 2, 5, and 6 were inserted polyaxially at a 10° angle off the transverse plane of the plate (Figure 3C). In both the second and third configurations, symmetry has been considered [17,21].

**Figure 4:**
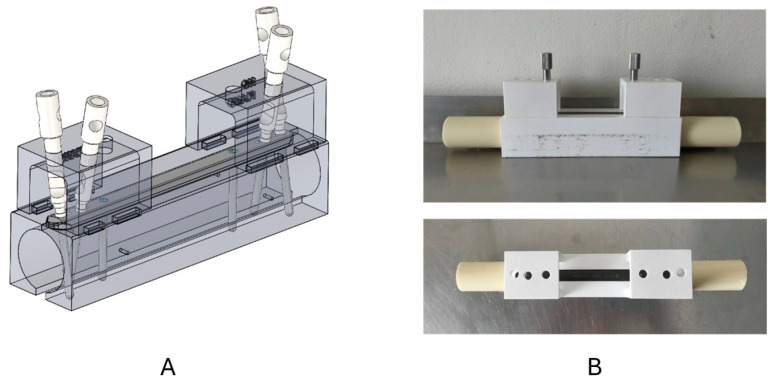
Custom drill guide designed to ensure the correct insertion of screws and the repeatability of each bone/plate/screw interface: (**A**) 3D CAD model (Group 3); (**B**) 3D printed model.

To ensure the correct insertion of screws and the repeatability of each bone/plate/screw interface, a drilling and plate fixation guide was designed and manufactured via 3D printing (Figure 4). The design of the mounting guide was tailored by adapting its geometry to the three-dimensional CAD models of the polyaxial locking plate system and the synthetic bone model. This system facilitated precise assembly, particularly in configurations involving polyaxiality. The mounting guide comprises 4 pieces. Two of these pieces embrace the synthetic bone and feature a groove on their upper part with geometry identical to that of the plate. This ensures that the plate’s position relative to the bone remains consistent in each assembly. The remaining two pieces fit into the first two above the plate. These two pieces each have three holes perfectly aligned with the plate’s holes. A pair of hole pieces was designed for each of the established configurations. Once all the pieces were assembled, the drilling guides supplied by the manufacturer of the polyaxial plate model used were employed to make each of the 2.8 mm diameter perforations on the synthetic bone. Subsequently, once the perforations were made at the desired angle, each screw was aligned with the previously created drilling guide and inserted into the synthetic bone. Each screw was inserted using a torque wrench supplied by the plate manufacturer, with a maximum tightening torque of 2 N·m.

### 2.5. Quasi-Static Tests

With the aim of defining the failure criterion for cyclic tests, a torsional quasi-static test was carried out. Quasi-static torsion tests were performed using a static servo-hydraulic testing machine. The load in the torsion tests was applied at a speed of 0.1 degrees per second until catastrophic failure occurred.

All constructs were adapted to the servo-hydraulic mechanical testing machine and the dynamic torsion testing machine using custom clamps. For this test, the first screw configuration was used (Figure 3A). The magnitude of the applied load and the actuator displacement were recorded at a frequency of 100 Hz. In all cases, the maximum load value obtained for each of the failure criteria was recorded.

### 2.6. Cyclic Tests

A total of 15 cyclic tests were performed. Each group was composed of five specimens. All constructs were adapted to the servo-hydraulic mechanical testing machine and the dynamic torsion testing machine (10 N·m, 0.5 mV/V) using custom clamps. The maximum force applied for compression and torsional tests was determined from preliminary static tests.

**Figure 5:**
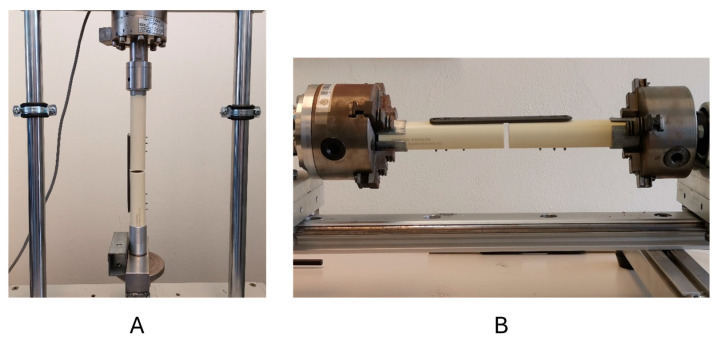
Mechanical testing machines using custom clamps to secure the surrogate bone: (**A**) axial compression test; (**B**) torsion test.

Each construct was tested sequentially in axial compression and torsion, in that order [31,32]. Figure 5 shows the set-up of both cyclic tests. In the cyclic compression test, each construct was positioned vertically, clamping the ends of the constructs with cylindrical jaws (Figure 5A). To simulate the joint formed by the hip and the femur, a custom jaw with a ball joint system was designed, upon which the load was applied. Initially, constructs were preloaded with 200 N. Then, a sinusoidal axial compression load ranging from 0 to 350 N at a frequency of 1 Hz was applied. Subsequently, torsion cycle testing was carried out.

In the cyclic torsion test, each construct was positioned horizontally, with the ends clamped using cylindrical jaws (Figure 5B), which applied the torque. On the opposite clamp of the testing machine, the surrogate bone cylinder had free axial movement to prevent axial load during the test. A fully reversed sinusoidal torsion load was applied to the constructs with a toque ranging from −4 Nm to +4 Nm at a frequency of 1 Hz [32,33]. Displacement, load, angle, and torque were recorded at a sampling frequency rate of 100 Hz by the traducers of the test system.

For the axial compression tests, the displacement data were recorded both in the near and far cortex. The initial data cycle was retained throughout the recording process and, and subsequently, data were recorded every 100 cycles up to 5000, at which point the tests were programmed to stop. The rotation angle was measured at the end of the surrogate bone for the torsion tests, where the testing machine generates the torque. The initial data cycle was retained, and then data were recorded every 100 cycles up to failure. The failure criterion was defined as a catastrophic failure due to fracture of the surrogate bone, loss of stiffness equal to 27% (±20°), or screw bending resulting in noticeable malalignment of the two bone segments.

### 2.7. Stiffness Assessment

The stiffness of each construct in all testing modes was determined from the slope of the load–deformation curve. For the axial compression test, system stiffness, or the maximum load–total displacement relationship was determined by the ratio F/δ (where F is the force applied by the machine in N and δ is the total displacement, from 0 to the current value, expressed in mm). For the torsion test, system stiffness was determined by the ratio M/θ (where M is the torsional moment applied by the machine in N·m at one of its ends and θ is the angle rotated by this end, expressed in degrees) [34].

### 2.8. Statistical Analysis

Tukey’s multiple comparison tests were performed to identify any significant differences between the means of the three groups for each of the following variables: stiffness after 5000 compression test cycles and the maximum number of cycles for the torsion test. A paired samples *t*-test was used to compare mean differences (among the three different configuration groups). The significance level was set at a probability error of 5% (*p* < 0.05). Statistical analyses were conducted using the software “Statistical Package for the Social Sciences” (SPSS version 18.0).

## 3. Results

### 3.1. Quasi-Static Torsion Tests

**Figure 6:**
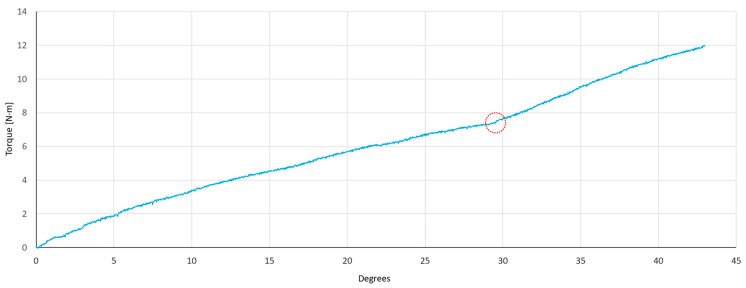
Quasistatic torsional test to define the failure criterion for cyclic torsion tests. First configuration of screws (Group 1).

Figure 6 depicts the results obtained from the quasi-static torsion test conducted to determine the failure criterion. It can be observed that as the torque increases, the stiffness of the plate decreases until reaching a value of 29.5 degrees of rotation, equivalent to 7.3 N·m of torque. Once this value is reached, the system’s stiffness increases again because the locking of the screw is lost, and the maximum rotational value is reached.

Upon analysis of the data and the tested specimen, it was determined that this phenomenon is due to the inherent characteristics of the locking system used by the polyaxial plate model under investigation in this research. This system consists of a spherical bushing that allows polyaxial movement and features a hole into which screws are inserted. When the tightening torque recommended by the manufacturer is applied, the bushing expands, thus locking the screw at the desired angle.

Because the locking mechanism relies on the principle of friction, when a torque value capable of overcoming this frictional force is reached, the screw locking system collapses, thereby losing its locking capacity and allowing the screws to vary their initial position, reaching the maximum allowable polyaxial limit in each hole. As the maximum polyaxiality value is reached, the stiffness of the screws to torsion increases, resulting in an increase in the slope of the graph depicted in Figure 6 (marked in red). 

### 3.2. Cyclic Tests

The first phase of cyclic tests involves pre-conditioning the assembly consisting of bone, plate, and screws through 5000 cycles of compression. Each tested specimen from each of the configured groups underwent this pre-conditioning.

**Figure 7:**
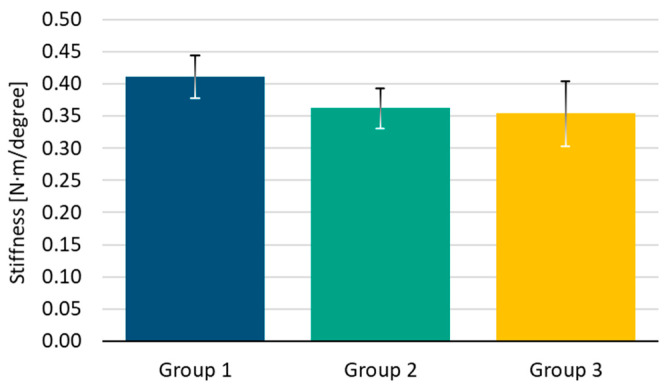
Initial torsional stiffness [N·m/degree] after 5000 of cyclic compression testing for each configuration.

**Figure 8:**
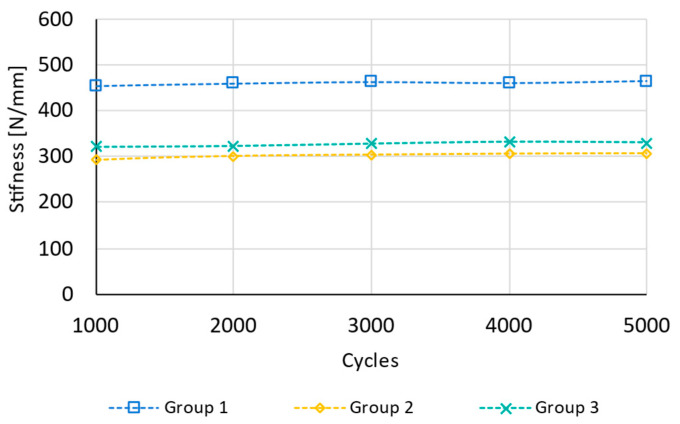
Stiffness variation [N/mm] of cyclic compression testing for one specimen of each group.

The results obtained during this phase are shown in Figure 7 and Figure 8. These figures primarily depict two different phenomena.

Firstly, the variation in stiffness after 5000 cycles of compression was analyzed. Group 1 achieved the highest initial stiffness with a mean value of 0.41 ± 0.03 N·m/degree, followed by Group 2 and Group 3 which achieved a mean value of 0.36 ± 0.03 N·m/degree and 0.35 ± 0.05 N·m/degree, respectively. Table 1 indicates the result of the Tukey test for initial stiffness. This analysis suggests that there were no significant differences between Groups 1 and 2 (*p* = 0.1 > 0.05, Table 1), 1 and 3 (*p* = 0.16 > 0.05, Table 1), and Groups 2 and 3 (*p* = 0.87 > 0.05, Table 1). This can also be observed in Figure 6, which represents the mean value of initial torsional stiffness obtained after 5000 cycles of compression loading. Group 1, where all screws were inserted without polyaxiality, exhibits the highest stiffness. Group 2, with screws at 10° of polyaxiality on the longitudinal plane of the bone, demonstrates the second highest stiffness prior to torsional fatigue testing, while Group 3 shows the lowest stiffness.

On the other hand, Figure 8 illustrates the evolution of stiffness for one specimen from each group during the 5000 cycles of compression performed prior to cyclic torsion testing. This figure corroborates the findings in Figure 7, showing that there is no variation in stiffness throughout the test in any of the configurations. This effect was observed in the remaining tested specimens as well.

**Figure 9:**
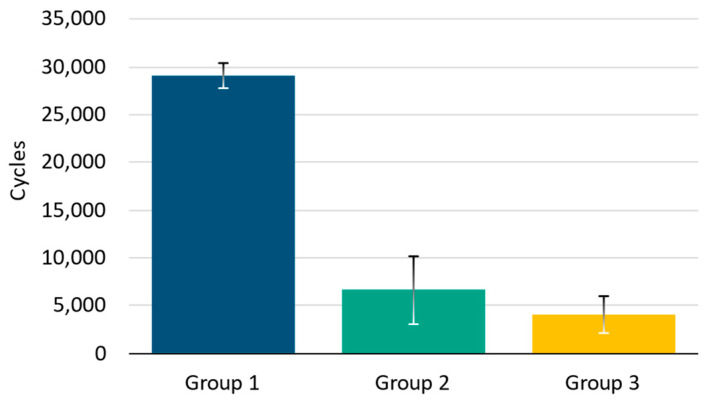
Mean number of cycles before failure and standard deviation of cyclic torsional testing for one specimen of each group.

**Figure 10:**
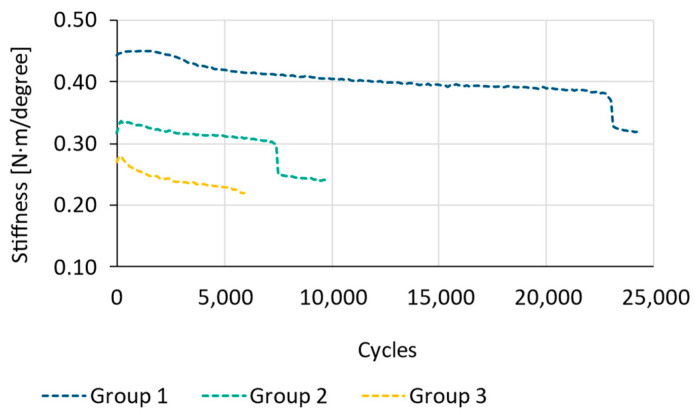
Stiffness variation [N·m/degree] of cyclic torsional testing for one specimen of each group.

The second phase of cyclic tests involved conducting a torsion test for each of the assemblies from the described groups until reaching the established failure criterion. Figure 9 displays the mean value of the maximum number of cycles reached and its standard deviation for each of the tested configurations. The results reveal that the effect of polyaxiality results in a considerable reduction in the maximum number of cycles achieved. This effect can be seen in Figure 10 which shows the stiffness variation [N·m/degree] of the cyclic torsional test for one specimen of each group. Group 1 achieved a mean value of 29,096 ± 1342 cycles, Group 2 a mean value of 6657 ± 3551 cycles, and Group 3 a mean value of 4085 ± 1934 cycles. This is also reflected in the results obtained in the Tukey test, which identifies differences between the various tested groups. The results presented in Table 1 show significant differences between the means of Groups 1 and 2 (*p* = 6.13 × 10^−5^ < 0.05) and Groups 1 and 3 (*p* = 1.83 × 10^−6^ < 0.05). However, there are no significant differences between Groups 2 and 3 (*p* = 0.26 > 0.05). This difference is clearly visible in Figure 8, which represents the mean maximum number of cycles and the standard deviation of the torsion analysis for each of the groups.

## 4. Discussion

The use of a narrow locking plate with six holes (three on each end) and polyaxial capability, which could offer the benefits of minimally invasive interventions along with the advantages of polyaxiality, was investigated. These advantages allow for the configuration of cross-screw fixation patterns, such as one analyzed in this study. This enables the surgeon to combine different prosthetic systems without interference and explore regions of higher bone density or unite multiple bone fragments. All these factors are crucial for ensuring proper patient recovery. However, despite the numerous advantages, these systems require greater skill and specific instruments to insert screws at the desired angle without deviations that may lead to complications. Therefore, it is necessary to complement commonly used drilling systems with drilling guides that facilitate the process and ensure the success of the procedure.

In order to ensure the repeatability of each tested specimen, a mounting system was developed in this research using 3D printing. This system was used to prepare the plate/screw/bone interfaces for each of the predefined screw insertion angles.

The mechanical properties of the plate/screw interface of different types of polyaxial locking systems have been extensively studied by various authors in previous research. J. E. Tidwell, 2016; J. Kaczmarek, 2022; J. Glowacki, 2023 analyzed the mechanical properties of different types of polyaxial locking mechanisms widely used in the recovery of complex bone defects. They inserted screws at angles of 0, 5, 10, and 15 degrees using a standard monoaxial locking mechanism. All of them concluded that as the polyaxial angle increases, the resistance to rotation of the screw/plate interface decreases almost linearly, with significant stiffness losses occurring with polyaxial angles above 10 degrees. For this reason, it was determined in this investigation that the maximum polyaxial angle used in the analyzed configurations would be 10 degrees [17,18,20].

The analysis conducted in this research focuses on studying the biomechanical properties of a fracture model with polyaxial plates under torsional loading conditions. The results of the tests conducted in this study are consistent with previous studies, indicating a common negative effect of polyaxiality on the locking system’s stiffness. Furthermore, this effect becomes more pronounced when the system is subjected to torsional stresses, which are prevalent in bones where large bone defects commonly occur, such as the femur, tibia, or fibula, and represent one of the most demanding loading conditions for locking systems. However, it is important to note that during the recovery process of a large bone defect, initially, the patient’s movement is restricted, significantly reducing the number of load cycles and the initial loads supported by the intervened region. This results in a progressive increase in the number of load cycles experienced by the plate/screw interfaces over time and a decrease in the loads supported due to the formation of bone callus. For this reason, it is important to note that the results obtained in this research are of great value in relation to the knowledge of the limits of the tested polyaxial locking plate, but cannot be directly transferred to clinical practice because the load has not been applied in a progressive manner and the decrease in the supported loads due to the formation of bone callus has not been considered.

Firstly, preconditioning was performed for each group, which involved subjecting the bone/plate/screw assembly to axial compression load for 5000 cycles. The results obtained for each group showed a common behaviour pattern during the initial phase of the tests. None of the groups experienced a loss of stiffness during the 5000 cycles of compression. However, differences in the mean stiffness values were observed among the tested groups, with Group 1 exhibiting the highest stiffness, followed by Group 3, which experienced 29% less stiffness than Group 1. Group 2 exhibited the lowest compression stiffness (35% less than Group 1). The absence of variations in stiffness during the 5000 cycles of compression allowed for the determination of the favourable behaviour of the plate model under compressive loads. One possible explanation for this phenomenon is related to the working length of the plate, which only has holes to insert screws at its ends, and the material of manufacture (Ti), which provides it with a lower Young’s modulus than other locking systems made of steel, thus avoiding plastic deformations or plate rupture failures. It is important to consider that a reduced elastic modulus of the bone/plate/screw assembly can result in significant movements in the fracture zone. From a theoretical perspective, a less rigid system may be advantageous in promoting bone callus formation in large bone defects, avoiding potential complications such as bone resorption or stress shielding, while excessive interfragmentary movement can lead to delays in the patient’s recovery process or cause non-union. However, the appropriate amount of interfragmentary micromovements is a factor that still requires further investigation and is conditioned by multiple factors [35,36].

Secondly, after the 5000 cycles of axial compression, each specimen was subjected to a torsion test until surpassing the established failure criterion. During this phase of the tests, significant differences were observed in the maximum number of cycles reached among the tested groups. In the case of compression tests, torsion stresses are considered negligible, and the fluctuating load applied (from 0 to 350 N) and the applied fluctuating load (from 0 to 350 N) never changed direction. Therefore, the bone/plate/screw assembly is always subjected to a negative load value. However, the cyclic load applied during torsion tests fluctuated between positive and negative torque values, causing changes in the direction of the stresses supported by the screws. This effect promoted screw loosening, and in some cases, total loss of locking, resulting in a significant decrease in system stiffness.

The results obtained showed that the configuration in which all screws were inserted orthogonally (Group 1) exhibited the highest initial torsional stiffness and supported the most load cycles compared to the other configurations. On the other hand, Group 2 and Group 3 showed very similar initial torsional stiffness values; however, Group 2 demonstrated greater resistance to torsional load cycles compared to Group 3.

The diversity of polyaxial locking systems and types of plate/bone/screw interfaces used in previous studies makes a direct result comparison a challenging task. M. Windolf et al. (2010) studied the biomechanical properties of a polyaxial configuration with cross screws on the longitudinal plane of the bone using a limited contact dynamic compression plate system (LC-DCP) [32]. The results obtained indicated a trend similar to that obtained in this research, with the polyaxial configuration achieving greater interfragmentary movements in both bending and torsion and lower stiffness values compared to a conventional LCP system. However, the results obtained by P. J. Denard et al. (2016) who analyzed the strength and stiffness of a biplanar locking system, with 9 degrees of polyaxiality on the transverse plane of the bone compared to a conventional locking system with screws inserted orthogonally, indicated that the biplanar locking system exhibited 42% higher stiffness than the orthogonal locking system [37].

Other authors have also analyzed the biomechanical properties of different polyaxial systems under bending and compression. J. Kaczmarek et al. (2020) conducted quasi-static bending and fatigue studies of an LCP system and a PLP system with a configuration of cross screws on the transverse plane of the bone. The LCP system achieved stiffness and strength values 42.5% and 48.6% higher, respectively, than those achieved by the PLP system. However, the maximum number of fatigue cycles reached was very similar [38]. This was also studied by B. W. Bufkin et al. (2013) who found higher stiffness in the PLP system and lower resistance compared to the results obtained by C.A. Blake et al. (2011) for an LCP system. On the other hand, a factor that still requires further investigation is the effect of polyaxiality in configurations where the plate is elevated above the bone surface [23,39]. In this regard, A. W. Tomlinson et al. (2015) analyzed the effect of multiple factors such as the number of screws, working length, plate elevation, and screw insertion angle of a PLP system. The results obtained indicated that the polyaxial configuration had slightly lower stiffness than its non-polyaxial counterpart. It was also observed that plate elevation above the bone surface resulted in a significant loss of stiffness (63%) and yield strength (63%) due to an increase in bending moment under axial load [21].

As is the case with the majority of studies, the present study is also subject to limitations that have prevented the inclusion of a more expansive range of configurations in the analyses. One of the primary constraints on the scope of this study has been the considerable financial investment required for the procurement of the surgical materials utilized in the mechanical tests. Furthermore, the inability to conduct in vivo mechanical studies has constituted an additional limitation, precluding the formulation of conclusions that can be extrapolated to actual clinical scenarios. Further research will include biomechanical studies incorporating flexion analysis, with the objective of providing a comprehensive description of the biomechanical behaviour of the polyaxial locking system proposed in this research. In addition, it would be beneficial to obtain the S-N curve, which would allow for the determination of the maximum number of cycles that can be supported by the polyaxial locking system as a function of an increasing load value. Consequently, further studies are required that reproduce real clinical situations, such as the combination of the locking plate system studied with intramedullary nails or in vitro analyses. This would facilitate the determination of the limits of the polyaxial locking system used, as well as the identification of the most suitable possible configurations for different types of clinical cases.

## 5. Conclusions

The biomechanical properties of a novel polyaxial plate system were investigated in this study. The results obtained showed that the system performed better when subjected to compressive loads than torsional loads.

In the case of compression tests, none of the configurations had a loss of stiffness after 5000 load cycles. This was verified by the *p*-values obtained with Tukey’s multiple comparison test, which established that there were no significant differences between the different configurations tested. However, it could be seen that the initial stiffness of the configuration in which the si bolts were inserted orthogonally (Group 1) was higher than that of the two configurations with polyaxiality (Group 2 and 3).

Torsional stresses are a major challenge for locking systems because they subject the bone/plate/screw assembly to torsional loading in different directions. These changes in direction generally lead to a tendency for locking systems to lose stiffness and, in many cases, to catastrophic failure in the form of screw loosening.

Finally, it has also been shown that, although polyaxiality has great advantages from a surgical point of view, it considerably limits the biomechanical performance of the system, causing a significant loss of rigidity. The results obtained show this phenomenon mainly in comparison with Group 1, which obtained much higher values of maximum torsional load cycles than those obtained by Groups 2 and 3, which did not show significant differences between them.

Polyaxiality has great advantages from a surgical point of view, it considerably limits the biomechanical performance of the system, causing a significant loss of stiffness. Torsional stresses are a major challenge for locking systems, because they subject the bone/plate/screw assembly to torsional loading in different directions. These changes in direction generally lead to a tendency for the locking systems to lose stiffness and, in many cases, to catastrophic failure in the form of screw loosening.

These phenomena could be observed in the results obtained in this study where it could be clearly seen that the configuration in which no polyaxiality was used showed a higher capacity in the maximum number of cycles achieved compared to the configurations in which polyaxiality was implemented. While Group 1 (all orthogonal screws) reached a mean value of 29,096 ± 1342 cycles, Groups 2 and 3 (four screws with polyaxiality) reached mean values of 6657 ± 3551 cycles and 4085 ± 1934 cycles, respectively. This could also determine that the non-linear distribution of the screws in the plate axis, although different from commercial plates commonly used with the aim of improving mechanical properties, does not prevent a loss of stiffness when using the polyaxiality.

The findings of this study demonstrate that polyaxiality still represents a significant challenge in terms of loss of stiffness. Nevertheless, the findings are of significant benefit to surgeons, providing insight into the mechanical behaviour of the polyaxial plate and its inherent limitations. This knowledge is invaluable in the decision-making process for optimal patient recovery.

## Figures and Tables

**Table 1:** *p*-values obtained with Tukey’s multiple comparison testing.

	*p*-Values after Torsion Tests	
	Group 2	Group 3
Initial stiffness		
Group 1	0.10	0.16
Group 2		0.87
Number of cycles before failure criterion		
Group 1	6.13 × 10^−5^	1.83 × 10^−6^
Group 2		0.26

## Data Availability

The data are contained within the article.
